# Relationships between job satisfaction, occupational burnout and rationing of care among intensive care unit nurses

**DOI:** 10.3389/fpubh.2024.1400169

**Published:** 2024-05-14

**Authors:** Katarzyna Tomaszewska, Krystyna Kowalczuk, Bożena Majchrowicz

**Affiliations:** ^1^Department of Nursing, Institute of Health Protection, The Bronisław Markiewicz Academy of Applied Sciences, Jarosław, Poland; ^2^Department of Integrated Medical Care, Medical University of Bialystok, Bialystok, Poland; ^3^Department of Nursing, Institute of Health Protection, State Academy of Applied Sciences, Przemyśl, Poland

**Keywords:** anesthesia nurses, care rationing, job satisfaction, occupational burnout, stress

## Abstract

**Introduction:**

Rationing of nursing care is referred to as overlooking aspects of required patient care. Its result is incomplete or delayed services provided to the patient. Anesthesia nurses employed in an intensive care unit are exposed to a significant workload. Particularly heavy is the psychological burden leading in many cases to the onset of burnout syndrome and a decrease in job satisfaction.

**The aim of this paper:**

Was to determine the relationship between occupational burnout, job satisfaction and rationing of care among anesthesia nurses employed in intensive care units.

**Materials and methods:**

The study group consisted of 477 anesthesia nurses employed in intensive care units in Poland. The study was conducted between December 2022 and January 2023. The research tools were BERNCA-R questionnaire, Job Satisfaction Scale questionnaire and Maslach Burnout Inventory questionnaire, which were distributed to selected hospitals with a request to be forwarded to intensive care units and completed. In the statistical analysis, correlations were calculated using Spearman’s rho coefficient, reporting the intensity of the relationship and its positive or negative direction. The analysis was performed using the IBM SPSS 26.0 package with the Exact Tests module.

**Results:**

The mean score of the BERNCA questionnaire was 1.65 ± 0.82. The mean score of occupational burnout was 60.82 ± 10.46. The level of emotional exhaustion, which significantly affects occupational burnout, was 26.39 ± 6.07, depersonalization was 14.14 ± 3.21 and lack of personal achievement was 20.29 ± 4.70. All the scores obtained exceeded the threshold of 50% of total points, which indicates the presence of occupational burnout at a significant level. The job satisfaction of the nurses surveyed was above mean at 23.00 ± 5.2 out of 35 total points.

**Conclusion:**

The results proved that there is a statistically significant, although with a weak strength of association, correlation between occupational burnout and rationing of care by anesthesia nurses. As the limitation of anesthesia nurses’ ability to perform certain activities increases, their job satisfaction decreases. In a work environment that is conducive to nurses, there are fewer job responsibilities that are unfulfilled. Therefore, it is essential to create a friendly work environment for nursing staff that will promote the provision of services at the highest possible level.

## 1 Introduction

Providing quality services is a priority activity for healthcare units, since good quality translates into health, trust and safety in terms of the patient’s life. The quality of care is considered mainly through the qualifications and competence of medical personnel, infrastructure and equipment base, as well as through the degree of patient satisfaction (1). Nurses, by virtue of their professional function, are in the closest contact with the patient. They are the ones who create the image of the facility and influence the climate of treatment (2, 3).

The concept of care overlooking is a fairly new phenomenon. It first appeared in 2006 introduced by Beatrice J. Kalisch and can be considered in any field of nursing. It refers to errors associated with the omission of all or part of a particular aspect of care (4, 5). The definition of care rationing in nursing refers to the deliberate failure to perform tasks resulting from patient care due to lack of time. Available publications prove that skipping care is directly related to the failure to ensure patient safety (6–9). The reasons for this phenomenon can be staff shortages, increased demand for care, new technologies and treatment methods, as well as greater patient awareness (10, 11). The totality of the aforementioned aspects translates into increased expenditures of time and labor incurred by nursing staff (12–14). The concept of care rationing according to Schubert et al. assumes that all nursing activities are equivalent, so that it is possible to achieve not only the desired activities, but also those expected by patients and the nursing diagnosis made, has a very strong impact on care rationing (15, 16).

Job satisfaction is understood as a higher level of job fulfillment. A satisfied employee has a higher sense of self-esteem and satisfaction of needs. In addition, work should be a place of self-fulfillment for employees; one in which they feel satisfied with their professional development and intellectual challenges. This, in turn, creates a sense of identification with the organization’s goals. Satisfaction, including job satisfaction, is also understood as an individual’s ability to realize their needs, goals, values, and beliefs (17).

Job satisfaction has been the subject of many studies on organizational behavior. Lu et al. (18) demonstrate that satisfaction on job satisfaction consists of several of various elements, including working conditions, communication, nature of work, organizational policies and procedures, pay and conditions, promotion opportunities, recognition, appreciation, safety, supervision and relationships. Although the description of job satisfaction varies, several common factors emerge in various studies, which include working conditions, organizational environment and perceived stress, role conflict and ambiguity, role perception and content, and organizational and professional commitment (19). Job satisfaction is considered a global problem; however, it is also necessary to improve the quality of care provided and the work environment in healthcare organizations, as lack of job satisfaction among nurses can affect their practice, which in turn can directly or indirectly affect patient satisfaction (20).

The nursing profession is associated with many stressful situations and a heavy workload. Contact with patients and their loved ones, lack of support from superiors often leads to psychotic symptoms, depression, sleep and wakefulness disorders in this professional group (21, 22). The causes of burnout can be traced to three areas: individual interpersonal and organizational. The above conditions may or may not promote occupational burnout (23). Occupational burnout syndrome usually begins unnoticed and the person affected often does not recognize the first symptoms. A common definition of the process is that it is a prolonged reaction to interpersonal and emotional stressors operating at work. Initially there is fatigue, irritability, tension, hyperactivity associated with symptoms of psychophysical exhaustion. As a result of the stress associated with the state of tension in the next phase, there is a loss of energy, discouragement and there are signs of depression related solely to the work situation (24). While studying the phenomenon of burnout, Maslach and Jackson distinguished two groups of variables: factors that can promote burnout and behaviors that help effectively combat burnout. The result of the study was the clarification of a characteristic picture for occupational burnout among nurses. This phenomenon increases with an increase in assigned tasks or a decrease in decision-making opportunities at work. These factors result in feelings of emotional exhaustion and a tendency to depersonalize patients. Depersonalization increases when feedback from patients decreases. According to Jackson and Schuler’s study, the risk of occupational burnout increases with the inability to control the environment. This finding was based on the premise that a rewarding environment for an employee is one that he or she can influence, while an unfavorable environment is one in which the ability to control is limited (25).

The work of the anesthesia nurse is one of the most difficult and responsible specialties in the profession due to its nature. Nurses working in intensive care units are particularly vulnerable to occupational burnout syndrome and belong to high-risk groups. The work, the essence of which is the care and nursing of a person in a life-threatening condition, such as after an accident, after surgery, in an advanced stage of illness or in a terminal state, exhausts psychologically primarily by constantly moving around final, critical issues, exhausts by its intensity (26).

Rationing of nursing care has a significant impact on the quality of services provided (22). Therefore, the authors set the aim of this study to investigate the relationship between occupational burnout, job satisfaction and rationing of care by anesthesia nurses employed in intensive care units. Analysis of such correlations will answer the question of whether the number of skipped activities by anesthesia nurses is lower in a psychosocially friendly work environment.

## 2 Materials and methods

### 2.1 Research design

In the present study, a cross-sectional survey was conducted among nurses employed in intensive care units in Poland who were providing work during the survey. The research instruments were a survey questionnaire consisting of questions on sociodemographic data and standardized research tools: BERNCA-R questionnaire (27), Job Satisfaction Scale (JSS) questionnaire (28) and Maslach Burnout Inventory (MBI) questionnaire (29). The study was conducted from December 2022 to January 2023. The innovation of the study is the evaluation of the relationship of socio-professional factors such as occupational burnout and job satisfaction with the level of care by anesthesia nurses. This is one of the first studies of this scope in the indicated group in Poland. The survey was conducted between December 2022 and January 2023. The innovation of the study was assessment of the impact of socio-professional factors such as occupational burnout and job satisfaction related to the level of care of anesthesia nurses. This is one of the first studies of this scope in the indicated group in Poland.

### 2.2 Research tools

#### 2.2.1 BERNCA-R care rationing questionnaire

The study used the Polish version of the BERNCA-R questionnaire adapted by Uchmanowicz et al. to assess rationing of care (27). The reliability of the translated version of the questionnaire was assessed using internal consistency and trustworthiness of the respondents. Cronbach’s alpha and inter-item correlations were used to analyze the internal consistency of the Polish BERNCA-R questionnaire. The mean total BERNCA-R score was 1.9 points (SD = 0.74) on the 0–4 scale. Cronbach’s alpha for the unidimensional scale was 0.96. The mean inter-item correlation was 0.4 (range 0.1–0.84), indicating high internal consistency. The univariate solution showed stable loadings above 0.5 for almost all items of the Polish BERNCA-R questionnaire. A study using the Polish BERNCA-R questionnaire showed that the tool is relevant and reliable for studying care rationing in groups of Polish nurses.

The questionnaire contains 32 questions about nursing activities rated on a 5-point Likert scale against which skipping care may have occurred. It is divided into five sections and respondents are asked to indicate how often in the past 7 days they were unable to perform them. The questionnaire score is the mean value of above-mentioned 32 questions. It therefore ranges from 0 to 4 and can be interpreted analogously to the interpretation of a single question. In addition, the questionnaire included questions on sociodemographic data: age, gender and job seniority of respondents.

#### 2.2.2 Job satisfaction scale (JSS)

The Job Satisfaction Scale (JSS) was used to measure overall cognitive job satisfaction. Zalewska constructed this tool based on the Satisfaction with Life Scale by Diener et al. (28). She reformulated the statements to address the sphere of work as a holistic, complex phenomenon and to require a conscious evaluation of work based on personal criteria. The scale is one-dimensional, consisting of five statements, rated on a seven-point scale. The JSS score is the total score of five questions.

There are no standard scores for low or high job satisfaction for this tool. The job satisfaction survey is based on 5 job-related statements, each rated on a 7-point scale: 1 – “strongly disagree,” 2 - “disagree,” 3 - “rather disagree,” 4 - “difficult to say,” 5 - “rather agree,” 6 - “agree,” 7 - “strongly agree.” A respondent can obtain between 5 and 35 points. The higher the score, the higher the perceived job satisfaction. The Job Satisfaction Scale measures the cognitive aspect of overall job satisfaction. The internal reliability of the scale is high in the heterogeneous group, Cronbach’s alpha is 0.864. JSS is a reliable tool for assessing overall job satisfaction, showing high consistency with similar scales.

#### 2.2.3 Maslach burnout inventory (MBI)

The Maslach Burnout Inventory (MBI) developed by Maslach et al. (29) is recognized as a standard tool for research in this field. The tool consists of three parts that examine emotional exhaustion, depersonalization and lack of personal achievement. The inventory consists of 22 items in the form of statements that are related to the attitudes and feelings the respondent feels about her professional work. The purpose of the questionnaire is to measure the intensity and frequency with which she experiences certain symptoms related to job burnout. The higher the final score, the more likely the respondent is to experience occupational burnout. The exception is in the area of personal fulfillment.

### 2.3 Participants

The study involved 477 anesthesia nurses employed in intensive care units. Inclusion criteria were work as an anesthesia nurse in an intensive care unit and consent to participate in the study. All questionnaires are physically stored by the authors of this paper. Paper questionnaire forms were distributed to 120 public hospitals in Poland with a request to forward them to intensive care units. The hospitals were selected from a list available on the Polish National Health Fund portal, while the authors had no influence or knowledge of which nurses would complete the questionnaires. The average number of nurses employed in these units depends on the number of beds and ranges from about 30 to 60. It was assumed that up to 20% of the anesthesia nurses employed in the selected hospitals would be surveyed. 600 paper survey questionnaires were initially distributed, 512 were returned and, after final verification, 477 correctly filled ones were subjected to statistical analysis (79.5%). The exclusion criteria were lack of consent to participate in the study, working in the pediatric intensive care unit, working one shift, working outside the intensive care unit.

### 2.4 Statistical analysis

In the statistical analysis, correlations were calculated using Spearman’s rho coefficient, reporting the intensity of the relationship and its positive or negative direction. The analysis was performed using the IBM SPSS 26.0 package (IBM, New York City, NY, United States) with the Exact Tests module. All correlations and differences are statistically significant when *p* < 0.05.

### 2.5 Ethical procedures

The participation of nurses in the study was voluntary and anonymous. The study was conducted in accordance with the ethical standards set forth in the Declaration of Helsinki (64th WmA General Assembly, Fortaleza, Brazil, October 2013) and in accordance with Polish legal regulations. The application was favorably approved by the Bioethics Committee of the State Academy of Applied Sciences in Przemysl (KBPANS 2/2023).

## 3 Results

The survey was conducted among 477 selected anesthesia nurses employed in intensive care units in Poland. 4.0% of the respondents were under 25 years old, 60.2% between 25 and 35 years old, 19.9% between 36 and 45 years old, and 14.9% were between 46 and 55 years old. Only 1.0% of respondents were over 56 years old. Respondents’ length of service as anesthesia nurses from 1 to 10 years was declared by 63.5% of respondents, from 11 to 20 years by 20.1% of respondents, 21 to 30 years by 12.6% and over 31 years by 3.8% of respondents. Detailed results are presented in Table 1.

**Table 1:** Characteristics of the study group.

Variable		Frequency (*n* = 477)	
Gender	Female	420	88.1%
	Male	57	11.9%
Age (years)	<25	19	4.0%
	25–35	287	60.2%
	36–45	95	19.9%
	46–55	71	14.9%
	>56	5	1.0%
Job seniority (years)	1–10	303	63.5%
	11–20	96	20.1%
	21–30	60	12.6%
	>31	18	3.8%

Nurses participating in the study worked 12-h shifts. According to the Polish Labor Code, there is a two-and three-month reference period (decided by the employer). As a rule of thumb, during the indicated period, the number of night and holiday duties for each nurse should be comparable.

### 3.1 Results of the BERNCA-R questionnaire

Statistical analysis showed that the most frequently rationed activities in the work of anesthesia nurses (the highest mean questionnaire score) during on-call were talking to the patient and/or his family (1.91 ± 1.18), lack of time to review individual patients’ situations and care plans at the start of the on-call period (1.86 ± 1.05), showing emotional or psychosocial support to the patient (1.84 ± 1.16), or giving patients sufficient information about upcoming tests or treatment (1.83 ± 1.15). The results may indicate that respondents pay more attention to the implementation of instrumental activities and overlook those related to psychological support.

The mean total score of the degree of nursing care rationing assessed by the BERNCA-R questionnaire was 1.65 ± 0.82 on a scale of 0 to 4 (where 0 means “there was no need for it” and 4 means “often”) and ranged between “never” or “rarely.” The median of the results obtained was also at the mean level and was 1.66 (Table 2).

**Table 2:** Average score of the BERNCA-R questionnaire (*n* = 477).

	Average	Median	Standard deviation	Minimum	Maximum
Rationing of nursing care BERNCA-R (0–4)	1.65	1.66	0.81	0.00	4.00

### 3.2 Results of the job satisfaction scale (JSS) questionnaire

Analysis of the data on the level of job satisfaction of the nurses surveyed (JSS) showed that it was above the mean level and was 23.00 ± 5.21, meaning that the majority of nurses were satisfied with their job at a level above average (Table 3).

**Table 3:** Average score of the MBI questionnaire (*n* = 477).

	Average	Median	Standard deviation	Minimum	Maximum
MBI Total (22–88 pts.)	60.82	62.00	10.46	30.00	88.00
Emotional exhaustion 1–9 (9–36 pts.)	26.39	27.00	6.07	9.00	36.00
Depersonalization 10–14 (5–20 pts.)	14.14	14.00	3.21	5.00	20.00
Lack of personal achievements 15–22 (8–32 pts.)	20.29	20.00	4.70	8.00	32.00

### 3.3 Results of the Maslach burnout inventory (MBI) questionnaire

The mean overall score of occupational burnout among the anesthesia nurses surveyed was 60.82 ± 10.46 (median 62.00). The maximum number of points was 88. According to Maslach’s statement, his indicates that the higher the score, the higher the occupational burnout in the surveyed group. The mean score of emotional exhaustion, which significantly affects the level of occupational burnout, was 26.39 ± 6.07 out of 36 points, depersonalization was 14.14 ± 3.21 out of 20 points and lack of personal achievement was 20.29 ± 4.70 out of 30 points. All the scores obtained exceeded the threshold of 50.0% of possible points, which indicates the presence of occupational burnout at a significant level. The results are shown in Table 3.

### 3.4 Correlations

Statistical analysis shows that respondents “never” or “rarely” ration instrumental activities, and the most frequently omitted ones are those relating to the patient’s mental sphere. There are statistically significant correlations between the level of occupational burnout and rationing of nursing care, which are characterized by insignificant strengths of association. Respondents with higher professional burnout in each area are characterized by greater inability to perform certain activities (Table 4).

**Table 4:** Correlations between MBI, rationing of nursing care and job satisfaction levels.

			MBI Total (22–88 pts.)	Emotional exhaustion 1–9 (9–36 pts.)	Depersonalization 10–14 (5–20 pts.)	Lack of personal achievements 15–22 (8–32 pts.)
Spearman’s rho	Rationing of nursing care BERNCA-R (0–4)	Correlation coefficient	0.204^**^	0.190^**^	0.149^**^	0.115^*^
		Significance (two-tailed)	0.000	0.000	0.001	0.012
		Frequency (*n*)	477	477	477	477

**Correlation significant at the 0.01 level (two-tailed).

*Correlation significant at the 0.05 level (two-tailed).

Greater inability to perform certain activities is associated with lower job satisfaction, which may confirm the fact that respondents are aware of skipping certain activities in patient care, which may cause them to have lower levels of satisfaction with their professional tasks and thus the overall level of fulfillment in the nursing profession. The correlation is statistically significant (*p* < 0.001) but the strength of the association was found to be insignificant (Spearman’s rho equal to−0.202) (Table 5).

**Table 5:** Correlations between job satisfaction and rationing of nursing care.

			Job satisfaction scale – JSS (5–35 pts.)
Spearman’s rho	Rationing of nursing care BERNCA-R (0–4)	Correlation coefficient	–0.202^**^
		Significance (two-tailed)	0.000
		Frequency (*n*)	477

**Correlation significant at the 0.01 level (two-tailed).

## 4 Discussion

The aim of this paper was to determine the relationship between occupational burnout, job satisfaction and rationing of care among anesthesia nurses employed in intensive care units. This is an innovative study of a group of nurses providing services to patients in life-threatening conditions requiring intensive treatment, supervision, and enhanced care. The results of our study showed that the mean total score of the degree of nursing rationing assessed by the BERNCA-R questionnaire was 1.65 ± 0.82 (below average), which may mean that the surveyed group is aware of the fact of responsibility for the health of patients. In studies by other authors conducted in a professional group of nurses, the mean BERNCA-R score was 1.55 ± 0.15, indicating that the frequency of care rationing ranged from “never” to “rarely” (30) and was similar to the results in the own research. Other studies have shown a higher frequency of nursing rationing among nurses working on surgical wards, with a mean score of 2.72 ± 0.86, and nurses working on conservative wards (2.08 ± 0.86) (22) and were similar to the own research.

In the Schubert et al. study, the mean BERNCA score was 57 (1.69 ± 0.571) and, according to the authors, meant that nurses had to ration at least one of the 32 tasks indicated in the questionnaire during the last 7 days of work, which is confirmed by our own study and those of other authors (22, 30, 31). A slightly lower level of rationing (1.38 ± 0.62) was shown by Uchmanowicz et al. study (32).

The mean overall burnout score among the anesthesia nurses surveyed was 60.82 ± 10.46, which indicates the presence of burnout in the study group. The mean score of emotional exhaustion, which significantly affects the level of professional burnout was 26.39 ± 6.07, depersonalization was at 14.14 ± 3.21 and lack of personal achievement was at 20.29 ± 4.70. This was presumably due to too much mental stress, responsibility for human lives and working under constant stress. A study by other authors conducted in a group of professional nurses showed that the mean score of occupational burnout was at 49.27 ± 19.76, exhaustion 63.56, depersonalization 37.2, lack of achievement 47.07 (30). In another study, the total MBI score was 38.14 ± 22.93. The individual components of occupational burnout yielded values of emotional exhaustion equal to 44.8, lack of personal achievement equal to 40.66, depersonalization equal to 28.95 (33) and differed from the results of the own research.

In the own research, the level of job satisfaction of the nurses surveyed was above average at 23.00 ± 5.2, which means that most nurses were satisfied with their jobs at a level above average. In the Uchmanowicz et al. study, the average score obtained by respondents was 11.71 ± 5.97, suggesting that respondents’ answers indicate a state between “dissatisfied” and “rather dissatisfied” with their work. Scores ranged from 7 to 18, with a median score of 11 (34), which has been confirmed by other studies (30, 35).

In the own research, there are statistically significant correlations between occupational burnout and rationing of nursing care, which are characterized by insignificant strengths of association. Greater inability to perform certain activities is associated with lower job satisfaction. The correlation is statistically significant (*p* < 0.001) but the strength of the association was found to be insignificant (Spearman’s rho = −0.202).

A study by other authors observed a statistically significant positive correlation between the BERNCA-R scale and MBI (*p* < 0.05), and a negative correlation between the BERNCA-R scale and JSS (*p* < 0.05). Independent predictors of the BERNCA-R scale were the emotional exhaustion of the MBI scale and the assessment of the impact of independence on job satisfaction (p < 0.05) (34). Similarly, Radosz-Kanwa et al. proved that BERNCA-R scale scores correlated statistically significantly and positively (*r* > 0) with two (of three) subscales of the MBI questionnaire: emotional exhaustion and depersonalization (*p* < 0.001) (31), which was confirmed by our own research. Rascu et al. (36) showed that bedside rationing of care refers to the use of clinical judgment by nurses to prioritize how they allocate their time and skills in providing health care to patients due to major time and staffing constraints or other organizational issues. The study by Tamayo et al. (37) found that nurses most often rationed care, support and supervision tasks. It was less common to omit other aspects of care. Identifying factors influencing rationing can help identify starting points for hospital policy reforms, as evidenced by our own research. Studies by many authors have found an overall increase in rationing of care activities, with documentation and social support being the most rationed (22, 38, 39).

Jaworski et al. (8) demonstrate that higher job satisfaction can reduce the risk of rationing nursing care, which is supported by other studies (40). White et al. found that some respondents often reported not being able to provide necessary care to patients (41). Other research suggests that workplace atmosphere and relationships partially mediate the link between organizational climate and occupational burnout (42). A nurse’s job satisfaction is related to the level of nursing care provided; higher levels of rationed care mean higher levels of occupational burnout and lower job satisfaction, resulting in general dissatisfaction among this professional group (43). There were other studies conducted that identified lack of time, inadequacy of staff, lack of team dynamics as possible factors that affect rationing of nursing care (44).

## 5 Conclusion

The results proved that there is a statistically significant, although with a weak strength of association, correlation between occupational burnout and rationing of care by anesthesia nurses employed in intensive care units. As the limitation of anesthesia nurses’ ability to perform certain activities increases, their job satisfaction decreases. Therefore, it is important to ensure that intensive care units are adequately staffed with nurses, as there are fewer job responsibilities that are unfulfilled in the work environment that is conducive to nurses.

## 6 Limitations of the study

The results of the survey have a solid basis; however, the study may have some limitations. The study involved a professional group of anesthesia nurses employed in intensive care units of different ages and job seniority. The sample size was non-representative of the situation in Poland, also due to bias in the selection of respondents, which may limit the generalizability of the results. The sample size was not large enough, which may limit the generalizability of the results. Further multi-center surveys are needed to generalize the results and implement recommendations for management.

## Data availability statement

The raw data supporting the conclusions of this article will be made available by the authors, without undue reservation.

## Ethics statement

The studies involving humans were approved by the Bioethics Committee of the State Academy of Applied Sciences in Przemyśl. The studies were conducted in accordance with the local legislation and institutional requirements. The participants provided their written informed consent to participate in this study.

## Author contributions

KT: Conceptualization, Data curation, Formal analysis, Investigation, Methodology, Project administration, Resources, Software, Supervision, Validation, Writing – original draft, Writing – review & editing. KK: Conceptualization, Data curation, Formal analysis, Investigation, Methodology, Project administration, Resources, Software, Supervision, Validation, Writing – original draft, Writing – review & editing. BM: Conceptualization, Data curation, Formal analysis, Investigation, Methodology, Project administration, Resources, Software, Supervision, Validation, Writing – original draft, Writing – review & editing.
